# Develop a vascularized neuroimmune organoid model for studying sporadic Alzheimer’s disease

**DOI:** 10.1002/alz.088501

**Published:** 2025-01-03

**Authors:** Ranjie Xu, Yanru Ji

**Affiliations:** ^1^ Purdue University, West Lafayette, IN USA; ^2^ Purdue University, Lafayette, IN USA

## Abstract

**Background:**

Alzheimer’s disease (AD) is the leading cause of dementia, affecting 50 million people globally. Current AD animal models mainly focus on familial or inherited AD. These models often carry the APP and PSEN gene mutations from familial AD patients, or introduce microtubule‐associated protein tau (MAPT) mutations, which can cause frontotemporal dementia but are not linked to AD. Notably, there are significant species differences between humans and animals, and most AD therapeutics that have shown success in animal models have failed in clinical, highlighting the development of more translational human‐centric models for studying AD, particularly sporadic AD.

**Method:**

In the current study, we developed a human‐induced pluripotent stem cell‐based vascularized neuroimmune organoids that contain multiple cell types in the human brain, including neurons, microglia, astrocytes, and blood vessels. We further challenge brain organoids with human AD postmortem brain tissues‐derived extracts to induce AD pathologies for studying sporadic AD. Finally, we applied this new model to test the effect of AD immunotherapy, such as Lecanemab.

**Result:**

AD brain extracts that contain pathological seeds, such as amyloid and tau, successfully induce multiple AD pathologies in neuro‐immune organoids, including 6E10+Aβ plagues, AT8+tau tangles, as well as neuroinflammation.

**Conclusion:**

The neuroimmune organoid model provides a unique opportunity to model sporadic AD in a human cell setting. This innovative model also facilitates AD drug screening, particularly for testing AD immunotherapy, such as antibody‐based AD treatment.

